# Red turpentine beetle primary attraction to (–)-β-pinene+ethanol in US Pacific Northwest ponderosa pine forests

**DOI:** 10.1371/journal.pone.0236276

**Published:** 2020-07-30

**Authors:** Rick G. Kelsey, Douglas J. Westlind

**Affiliations:** United States Department of Agriculture, Forest Service, Pacific Northwest Research Station, Corvallis, Oregon, United States of America; USDA Agricultural Research Service, UNITED STATES

## Abstract

Red turpentine beetle, *Dendroctonus valens* (Coleoptera: Curculionidae: Scolytinae) is a non-aggressive pine bark beetle native to North America, and more aggressive invader in China. Dispersing pioneer beetles are attracted to potential host trees by oleoresin monoterpene kairomones, but respond more strongly to those combined with ethanol, a mixture often released from stressed, dying, or recently dead trees. (+)-3-Carene, usually the dominant or co-dominant monoterpene in ponderosa pine, *Pinus ponderosa*, is a stronger attractant than α-pinene or β-pinene where tested over a large portion of the *D*. *valens* range, while (+)-3-carene+ethanol was shown previously to attract twice the beetles of (+)-3-carene. A field test comparing *D*. *valens* attraction among the three monoterpenes when all are released with ethanol has never been reported, and was our objective. In three US Pacific Northwestern pine forests, (–)-β-pinene+ethanol lures attracted 1.4 to 1.9 times more beetles than (+)-3-carene+ethanol. (+)- or (±)-α-pinene+ethanol lures were least attractive. A 1:1:1 monoterpene mixture+ethanol lure attracted more beetles than the 1:1:1 lure, but it was not statistically higher. Monoterpenes were dispensed from low density polyethylene bottles and their release rates monitored in laboratory and field tests. Under laboratory conditions (+)-3-carene was released much more rapidly than (+)-α-pinene or (–)-β-pinene when dispensed separately, or in a 1:1:1 mixture. (+)-3-Carene in the 1:1:1 mixture increased the release of both pinenes over their rates when dispensed separately. (–)-β-Pinene+ethanol is currently the strongest kairomone lure for *D*. *valens* attraction in US northwest pine forests, and has value for beetle detection, monitoring, research, and management.

## Introduction

Red turpentine beetle, *Dendroctonus valens* LaConte, is a widespread species with endemic populations in pine and mixed conifer forests of the US−except southern Gulf Coast states, Canada, Mexico and Central America [1]. They attack all pine species across their native range and respond to a variety of host kairomones [2–7]. Although females produce frontalin, a dual function sex and aggregation pheromone [8], they are non-aggressive and normally do not mass attack healthy undamaged trees, relying instead on stressed, dying, or recently dead hosts [1]. Interest in *D*. *valens* host tree detection, selection, and kairomone primary attraction in the US has been stimulated by studies focused on bark beetle colonization of fire injured trees and their impact on post-burn tree mortality [9–21]. In China, interest in *D*. *valens* followed its establishment, population eruption and involvement in the death of *Pinus tabuliformis* Carri**ère** in large numbers [reviews 22, 23].

Anywhere from 4% to 40%, or more, of fire injured pine may be attacked by *D*. *valens* in western US forests [9, 10, 13–15, 18, 21], and as high as 60% of burned *P*. *tabuliformis* at one site in China [24]. Some attacks begin within days or weeks [9, 20], with most occurring in the first two years post-fire [11, 13, 15, 17, 18, 21]. They attack trees across a wide range of scorch height injury to the bole, and most attacked trees with light to moderate scorch survive, as *D*. *valens* colonization does not substantially increase their mortality [1, 13, 14, 18, 21, 25]. However, a portion of surviving trees are at risk to disease infection, since the external symbiont community vectored by *D*. *valens* may include forest pathogens such as *Leptographium wageneri* (W.B. Kendr.) M.J. Wingf. var. *ponderosum*, the cause of black stain root disease in ponderosa pine, *Pinus ponderosa* Douglas ex P. Lawson & C. Lawson [26], and bluestain from *L*. *terebrantis* Barras & Perry, among others [27, 28]. Living, diseased trees also attract *D*. *valens*, particularly if wounded [29, 30], and may function as disease redistribution centers when beetle offspring carry pathogen spores to other stressed trees in the surrounding forest. The availability of fire-injured host pines for *D*. *valens* has risen greatly in recent decades with increases in large wildfires, and annual area burned, especially in western US forests [31–34]. Trees are also injured by prescribed fire used to manage fuel loads and mediate severity of wildfires [35]. Annual area burned by wildfire is likely to remain high, or increase in the future, due to rising temperatures and drought [36–38], further expanding abundance of stressed host trees. While this increases the probability of native forest pathogen infections, whether it accelerates the spread of any mentioned above, remains unknown.

With such large numbers of fire stressed host trees, inadvertent reintroduction of *D*. *valens* into North America from China creates substantial risk for establishment and rapid distribution of any invasive pathogens they carry in their microbial symbiont community. Ten of the fourteen Ophiostomatalean fungal species isolated from beetles in China were not detected on *D*. *valens* collected in US forests [27]. *Leptographium procerum* (W.B. Kendr.) M.J. Wingf, the cause of Leptographium root disease, is the most common fungi isolated from beetles in China [24, 39], and is also present as a weak pathogen in the eastern US, but not known to occur in western US pine forests [27, 39, 40] where it poses a potential threat if introduced. Virulence for any of these fungi toward western pines is unknown, but expected to vary by fungal species, and strains within species as demonstrated with pine in China [24, 41]. In addition, fire stressed pine may have reduced resistance to these fungi, as lesions were larger in burned *P*. *tabulaformis* than in unburned trees for nearly all fungal isolates tested across four *Leptographium* species in China [24].

Kairomone lures have been used to detect, monitor, or manipulate invasive *D*. *valens* populations in China [42], and would be an important tool for managing reintroduced beetles to North American forests carrying novel forest pathogens. 3-Carene, α-pinene and β-pinene have been the focus of most *D*. *valens* kairomone attraction studies, as each is a dominant component of ponderosa pine oleoresin depending on geographic site [43, 44]. In one California study, (−)-β-pinene lures attracted 10.7 times more *D*. *valens* than (+)-3- carene that had beetle numbers similar to (+)-α-pinene lures [5]. Each increase in the (−)-β-pinene release rate was accompanied by a stronger beetle response, and the only study with this result. Elsewhere, (+)-3-carene attracted more beetles than (−)-β-pinene, (+)-α-pinene, or their 1:1:1 mixtures at test sites in California, Wisconsin, Pennsylvania, Mexico and China [6, 7]. While (+)-3-carene is the strongest *D*. *valens* lure in the US and China, healthy *P*. *tabuliformis* in China produce α-pinene as the major oleoresin monoterpene, with varying proportions of β-pinene, limonene, or myrcene, depending on tree diameter, but all with minimal (+)-3-carene [45, 46]. Large diameter (30 cm) *P*. *tabuliformis* contain α-pinene as the dominant component and attracts greater numbers of *D*. *valens* than smaller trees (10 cm) with nearly equal proportions of α-pinene and β-pinene in their oleoresin [45]. Furthermore, in laboratory olfactometers, bark volatiles from the large trees, or a comparable mimic blend of bark monoterpenes prepared artificially, were more attractive to *D*. *valens* than bark volatiles from small trees, or a mimic blend [45]. Clearly, various pine monoterpenes can function as primary attractants for *D*. *valens* when (+)-3-carene is absent, or minimally available.

Tree tissues synthesize and accumulate ethanol in response to various stressors including fire [20, 47, 48]. It functions as a physiological kairomone, rather than a host-specific oleoresin kairomone for *D*. *valens* and other bark or ambrosia beetles to detect stressed trees [49–51]. Ethanol by itself is a weak *D*. *valens* attractant [2, 52, 53], but when mixed with monoterpenes in stressed conifer tissues and released into the atmosphere [54] there is a stronger attraction. Ethanol concentrations in phloem and sapwood 2 cm above *D*. *valens* gallery entrances in fire damaged ponderosa pine were greater than in tissues from opposite sides of the same tree without a nearby gallery, and in tissues from unattacked neighbors with similar visual fire damage [20]. The *D*. *valens* attraction to (+)-3-carene+ethanol in an Oregon forest was confirmed as a synergistic response [53]; it attracted 2 times more beetles than (+)-3-carene, and 4 times more than ethanol. In Wisconsin, traps baited with a 1:1 (ethanol:turpentine) mixture caught 60 times more *D*. *valens* than traps baited with turpentine alone in preceding years [3]. Lures with high release β-pinene+ethanol attracted *D*. *valens* at a New York Christmas tree plantation, but was not compared with β-pinene lures [55]. In California, traps baited with 1:1:1 [(+)-α-pinene:(−)-β-pinene:(+)-3-carene]+ethanol lures captured 1.2 times more *D*. *valens* than traps with 1:1:1 lures, but the increase was not statistically greater [56]. Relative proportions and release rates of the lure components will likely impact the responses as observed for other beetles [57]. Lures with high ethanol release rates attracted 3.3 times more *D*. *valens* than those with low release when each was combined with 1:1 (α-pinene:β-pinene) in a central Oregon forest [58].

*Dendroctonus valens* is a host generalist [59] colonizing many pine species with variable proportions of α-pinene, β-pinene and 3-carene. While they respond most strongly to 3-carene when all are available, their attraction to 3-carene+ethanol is much greater [53], but how its compares to α-pinene+ethanol and β-pinene+ethanol is unknown. The objective of this study was to test *D*. *valens* primary attraction to (+)-3-carene+ethanol, (−)-β-pinene+ethanol, and (+)-α-pinene+ethanol or (±)-α-pinene lures in US Pacific Northwestern pine forests. At a fourth location their attraction was tested to a 1:1:1 mixture and 1:1:1 mixture+ethanol. Low density polyethylene (LDPE) bottles were used to release the monoterpene, as in previous studies by others. Release rate of each compound, and various mixtures were monitored in laboratory tests and used to adjust the number of bottles needed to provide similar release rates for field lures also monitored for each test.

## Materials and methods

### Ethics statement

This research was conducted on publicly owned and managed forests, with access provided by local land managers. No endangered or protected species were involved in this work.

### Study sites

**Fig 1:**
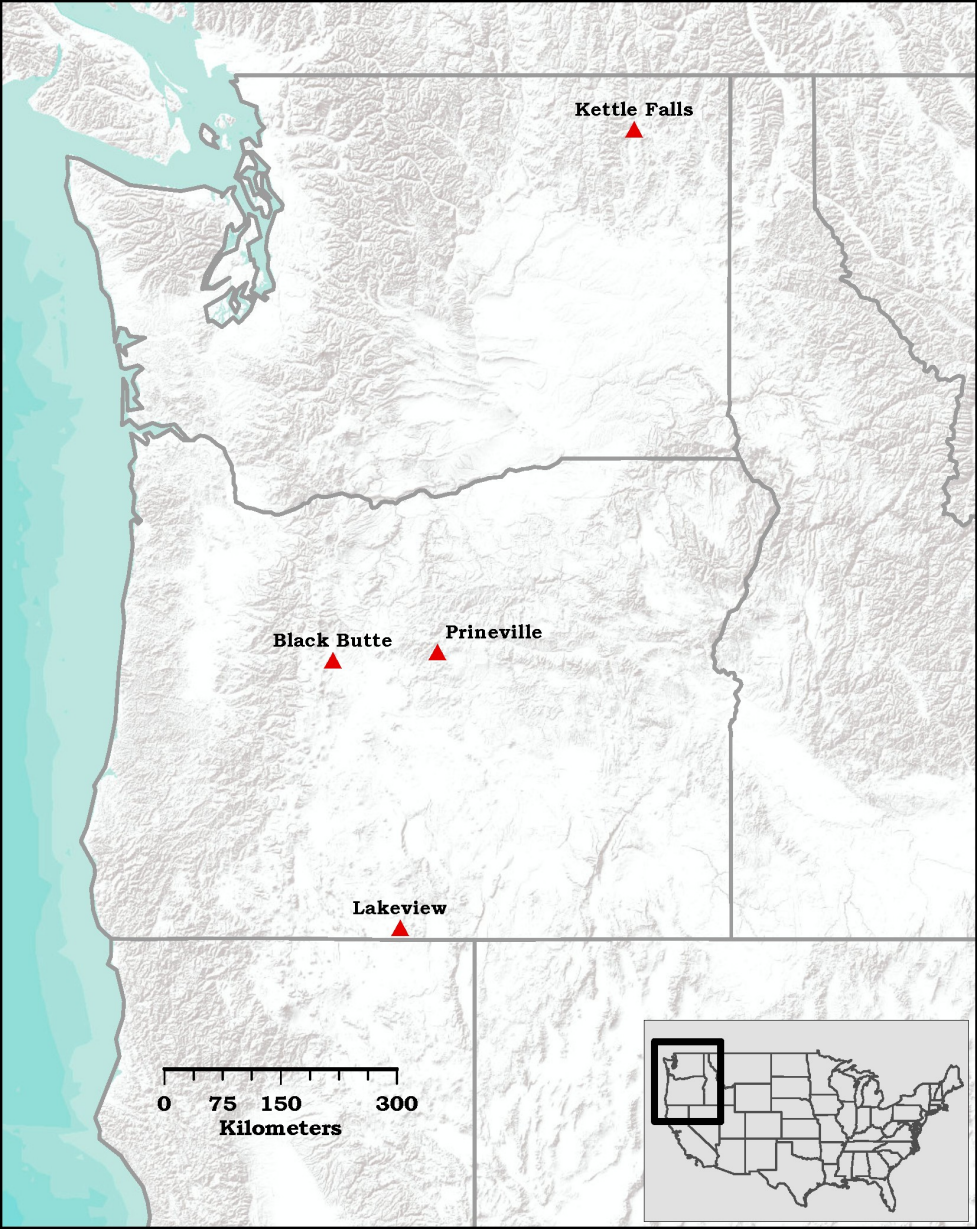
The four study site locations in Oregon and Washington, USA.

Field trapping tests were conducted on four widely separated sites (Fig 1) treated with prescribed fire within the previous year, where *D*. *valens* would be more abundant. Black Butte (44°21’05”N, 121°38’20”W, elevation 1015 m) is approximately 9 km aerial distance northwest of Sisters, Oregon (OR), Kettle Falls (48°35’21”N, 118°08’45”W, elevation 510 m) is about 5 km aerial distance southwest of Kettle Falls, Washington, Lakeview (42°05’24”N, 120°51’39”W, elevation 1725 m) is roughly 43 km aerial distance southwest of Lakeview, OR, and Prineville (44°25'15"N, 120°25'40"W, elevation 1450 m) is about 37 km aerial distance northeast of Prineville, OR (see S1 Appendix for detailed descriptions of all sites).

### Lures

**Fig 2:**
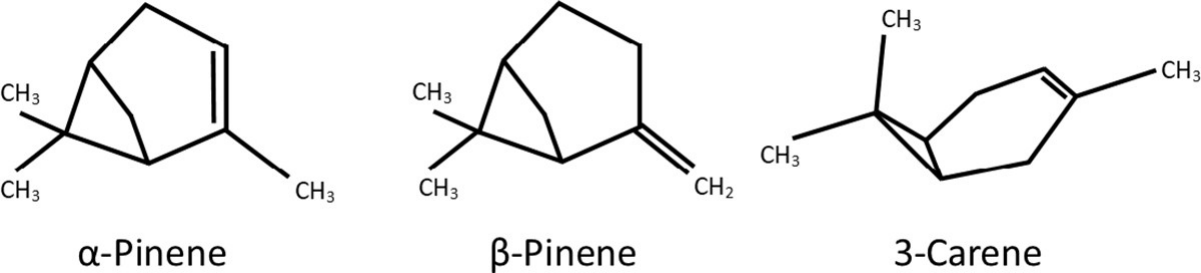
Structures of the three monoterpenes compared for primary attraction of *Dendroctonus valens* when released with ethanol.

Monoterpenes for Kettle Falls and Lakeview lures were (+)-α-pinene (97%), (−)-β-pinene (97%), and (+)-3-carene (> 90%) (Sigma-Aldrich Co., St. Louis, MO, USA; Fig 2 for structures). They were released from low density polyethylene (LDPE) bottles (15 ml, The Cary Company, Addison, IL, USA); filled to the threaded neck, leaving a small air bubble under the cap. Ethanol (95%, Synergy Semiochemicals Corp., Burnaby, Canada) was released from membrane pouches (approx. 59.6 x 8.4 cm inseams). To maximize vapor mixing the LDPE bottles and ethanol pouches were combined in a wide mouth high density polyethylene jar (9.3 W x 9.7 cm L cap on, 473 ml volume; Uline, Pleasant Prairie, WI, USA). Five equally spaced ventilation holes were drilled on both the jar bottom (0.79 cm) and the screw cap (0.95 cm) in an X pattern. A screen was attached inside the cap surface to prevent insects from entering through top holes, and the outside jar wall was wrapped with aluminum foil to reduce solar heating. One ethanol pouch was wrapped around the inside wall of the mixing jars leaving a center opening where LDPE bottles were placed, either one bottle of (+)-3-carene, or three bottles of (+)-α-pinene or (–)-β-pinene, to provide similar release rates based on laboratory tests.

Black Butte lures differed somewhat from Kettle Falls and Lakeview. A racemic mix of (±)-α-pinene (95%) instead of (+)-α-pinene (97%), and (−)-β-pinene (97%) were from Synergy Semiochemicals in LDPE bottles different from those used at Kettle Falls and Lakeview. Extra Synergy bottles were obtained to dispense (+)-3-carene (> 90%, Sigma-Aldrich). Ethanol pouches and mixing jars were the same as Kettle Falls and Lakeview except each pouch surface area was reduced by folding lengthwise at the middle, dividing ethanol equally into each side when held vertical. Excess membrane on top was folded repeatedly downward and secured with a pinch clamp leaving about 8.5 x 8.4 cm surface area on each side filled with ethanol. One bottle of (+)-3-carene, or three bottles of (±)-α-pinene or (–)-β-pinene were combined with one ethanol pouch in each mixing jar.

Ethanol pouch and LDPE bottle release rates were measured by weighing before initial placement into mixing jars, and after their return from the field. Ethanol pouches were stored in a 5°C coldroom in plastic bags as received from the supplier. Prior to weighing, they were unfolded in the laboratory to evaporate condensed ethanol while adjusting to room temperature. Immediately after weighing they were returned to the coldroom and placed in mixing jars later that day. Full LDPE bottles were immediately transferred to the coldroom after their initial weighing, grouped together by type and held in plastic bags until placed in mixing jars. After filling mixing jars with lures, they were separated by lure type into coolers and held overnight in the coldroom. Dry ice was added to coolers for transport to and from the field. Mixing jars were attached to traps outside the 6^th^ funnel above the catch cup. Upon return to the lab, coolers were stored in the 5°C coldroom until the next day when pouches and bottles were removed from mixing jars and sorted into groups. After wiping dust from surfaces of pouches and bottles, they were transferred to the laboratory for a set time allowing adjustment to room temperature before weighing.

The Prineville trapping test compared *D*. *valens* attraction to 1:1:1 [(+)-α-pinene:(−)-β-pinene:(+)-3-carene] mixed lures and 1:1:1+ethanol lures using the same LDPE bottles, monoterpenes and ethanol described for Kettle Falls and Lakeview, but with differences in the mixing jar size, number of ethanol pouches, and number of replicates for each lure type, with further details described in the S2 Appendix.

### Traps and field tests

Black Butte, Kettle Falls and Lakeview field tests had sixty, 16-unit multiple-funnel traps setup in 15 blocks as a randomized complete block design, with four randomly assigned lures (α-pinene+ethanol, β-pinene+ethanol, 3-carene+ethanol, and a no lure blank) per block. Blocks were a minimum of 50 m apart, and traps within blocks a minimum of 50 m apart, setup in a line at least 3 m from any potential host trees. Traps were secured to a metal rod bent 90^o^ at the top for attachment, with catch cup bottoms 10–30 cm above the forest floor depending on slope. Ethylene glycol with diethylene glycol antifreeze (Prestone Products Corp., IL, USA) was added to the catch cups to kill and preserve beetles [60]. Blocks were often positioned in groups to keep them within the burn perimeters and accommodate topographic features. For example, the Lakeview burn wrapped around a mountain, with a group of 8 blocks on the north side and another group of 7 blocks on the southeast side.

Black Butte lures were attached to traps on 29 April, and beetles collected 13 and 27 May and 9 June, 2016 (41 trap days). Lakeview lures were attached to traps on 5 May, with beetles collected 23 May and 6 June, 2017 (32 trap days). Kettle Falls lures were attached to traps on 17 May, with beetles collected on 1 and 15 June, 2017 (29 trap days). The *D*. *valens* were sorted from other trapped insects and counted. A 10% beetle subsample from each trap was randomly selected for gender determination based on morphology of the seventh abdominal tergite [61]. Due to low numbers, gender was determined for 100% of *D*. *valens* in the blank traps at each site.

Beetle responses from the three field tests were analyzed together using a randomized complete block ANOVA design with three lure types and three sites. Block (within site) was modeled as a random effect, while site and lure type were modeled as fixed effects. The total number of *D*. *valens* caught in each trap was summed over the entire period then normalized to number of beetles trapped/200 mg monoterpene released from the trap, to adjust for different monoterpene release rates and total trap days across sites. Beetles in blank traps without lures could not be normalized to monoterpene release rates, so the numbers caught/trap/day were analyzed by ANOVA with site modeled as a fixed effect. In both analyses, the beetle numbers were square root transformed to correct for model assumptions, with least squares means and 95% confidence intervals back-transformed for presentation. Female proportions in traps with monoterpene lures were adjusted by adding +0.0001 prior to square root transformation and analyzed by ANOVA using the same randomized complete block design above. Blank trap female proportions were not analyzed statistically. Daily monoterpene and ethanol release rates were analyzed separately using the same ANOVA design as the beetle catch above, but without transformations.

### Field lure temperatures

Temperatures the field lures experienced inside mixing jars were recorded with ibutton temperature data loggers (Maxim Integrated Corp. San Jose, CA, USA). At Black Butte, every jar had two ibuttons taped on the inside surfaces opposite one another to evaluate differences within and among jars. At Kettle Falls and Lakeview, one ibutton data logger was randomly assigned to eight mixing jars of each lure type, or 24 total, inserted between the ethanol pouch and inner jar surface.

Daily average, maximum, and minimum temperature in mixing jars were analyzed using a completely random ANOVA design. Each was compared among lure types at Black Butte with lure modeled as a fixed effect. They were compared also in jars among the three sites with site modeled as a fixed effect. No transformations were needed for any of the temperature analyses. The ibuttons at Kettle Falls and Lakeview were near the end of their battery life and a few stopped recording, leaving data from 22 loggers at Kettle Falls and 23 at Lakeview, so the data for Black Butte temperatures was randomly selected from 23 of the 45 lure jars.

### Laboratory experiments

In all laboratory experiments the monoterpenes and LDPE bottles were the same as for Kettle Falls and Lakeview lures above. They were positioned in a hood with exhaust fan off and safety window down to minimize air movement. Air temperature was monitored with three ibutton temperature data loggers. Building renovation contributed to higher and more variable laboratory temperatures than normal.

#### Monoterpene evaporation

Evaporation rates for (+)-α-pinene, (−)-β-pinene, (+)-3-carene, and a 1:1:1 mixture were determined by weighing ten glass vials (4 ml, 15 x 45 mm) for each compound filled to the threads at their neck base. They were weighed and analyzed as 10 blocks with one vial of each monoterpene. When placed in the hood, vials with similar contents were sorted together and separated by cardboard barriers to minimize any cross influences. They remained open for 17 days and reweighed. Evaporation rates of each monoterpene and their 1:1:1 mixture were compared using a randomized complete block ANOVA. Quantities released for each liquid were the response variables, with monoterpene type modeled as a fixed effect and block modeled as a random effect.

#### Monoterpene release from LDPE bottles

LDPE bottle release of (+)-α-pinene, (−)-β-pinene, or (+)-3-carene was monitored with ten replicates for each compound. They were weighed and analyzed as 10 blocks with one bottle of each monoterpene per block. In the hood, bottles were grouped by monoterpene type on a wire rung shelf in zones separated by 3 cm thick closed cell foam barriers to minimize any cross influences. Within zones the bottles were laid on their side in two rows 5 cm apart. They were reweighed daily for 21 days to determine 24 h, and cumulative release. After each weighing the bottle groups were shifted to the adjacent shelf zone, to minimize any position effect. The three monoterpenes 24 h release rates, or cumulative totals at day 1, 7, 14 and 21 were analyzed using a repeated measures ANOVA. Monoterpene type, day and their interaction were modeled as fixed effects and block (bottle, *n* = 10) as a random effect, with day repeated.

LDPE bottle release of a 1:1:1 [(+)-α-pinene:(−)-β-pinene:(+)-3-carene] mixture was monitored in two experiments. For the first experiment, 10 full weighed bottles were placed in the hood for 42 days and reweighed every 24 h. Bottle contents were sampled at day 0, 7, 14, and 42 for analysis by gas chromatography (GC) to determine proportional changes of each monoterpene over time. Proportions of each monoterpene remaining in the LDPE bottes at day 0, 7, 14, and 42, and their separate cumulative release at these same days were each analyzed as a randomized complete block, repeated measure ANOVA. Proportions, or cumulative release of individual monoterpenes were the response variables, with monoterpene type, day, and their interaction as fixed explanatory effects, block (bottle, *n* = 10) as a random effect, and day repeated.

In the second experiment, we examined the cumulative releases of 1:1:1, 1:4:1, or 1:1:3 mixtures and their individual monoterpene components using 10 LDPE bottles of each solution, as these mixtures have been tested as lures [6] and provided additional insight on how each monoterpene is released. They were processed in blocks and weighed daily over 21 days as described for the LDPE experiment above releasing individual monoterpenes. All bottles were sampled at day 0 and 21 for GC analysis to determine proportions of each monoterpene remaining to calculate their release. Cumulative releases of each mixture at day 1, 7, 14 and 21 were analyzed together as a randomized complete block, repeated measure ANOVA. The mixture type, day, and their interaction were fixed explanatory effects, block (bottle, *n* = 10) was a random effect, with day repeated. Individual release of (+)-α-pinene, (−)-β-pinene and (+)-3-carene at day 21 was compared for each mixture in separate randomized complete block ANOVA’s with monoterpene type modeled as a fixed explanatory effect, and block (bottle, *n* = 10) modeled as a random effect.

### GC analysis

In both experiments with mixtures, at designated sample dates one μl of solution was removed from each LDPE bottle with a syringe and transferred to a 1.5 ml GC autosampler vial containing 1.0 ml of hexane (HPLC grade, EMD Chemicals, Inc. Gibbstown, NJ, USA). All GC analyses were performed on a Hewlett Packard (HP, currently Agilent, USA) 5890 Series II gas chromatograph (GC) with a DB-5 column (30 m×0.25 mm, 0.25-μm film thickness, J&W Scientific from Agilent, USA) connected to a flame ionization detector using helium as the carrier gas at 1.0 ml/min set at the column oven starting temperature with a 1:20 split. The injector and detector temperatures were 250°C. One μl of each sample was analyzed starting at 100°C with no hold, then 3°C/min to 115°C, and finally 20°C/min to 260°C and no hold.

Samples of phloem were taken from 10 trees at Kettle Falls and Lakeview to examine the major monoterpene components using the same instruments above. Extraction procedures and GC parameters for their analyses are presented in the S3 Appendix.

### Statistical analysis

All statistical analyses described with each experiment were completed using the MIXED models procedure in SAS 9.4 [62]. Assumptions of normality and equal variance of residuals were checked during all analyses using quantile-quantile, and residual vs predicted plots, respectively. An unstructured UN(1) covariance structure was chosen for all repeated measures analyses. ANOVA results are presented in the figures. Treatment means comparisons were made by Fisher’s protected least significant difference; *P* values were considered statistically significant at *P* ≤ 0.05, and those with *P >* 0.05 < 0.10 marginally significant, but the latter mentioned only when considered relevant.

## Results

### Beetle catch

**Fig 3:**
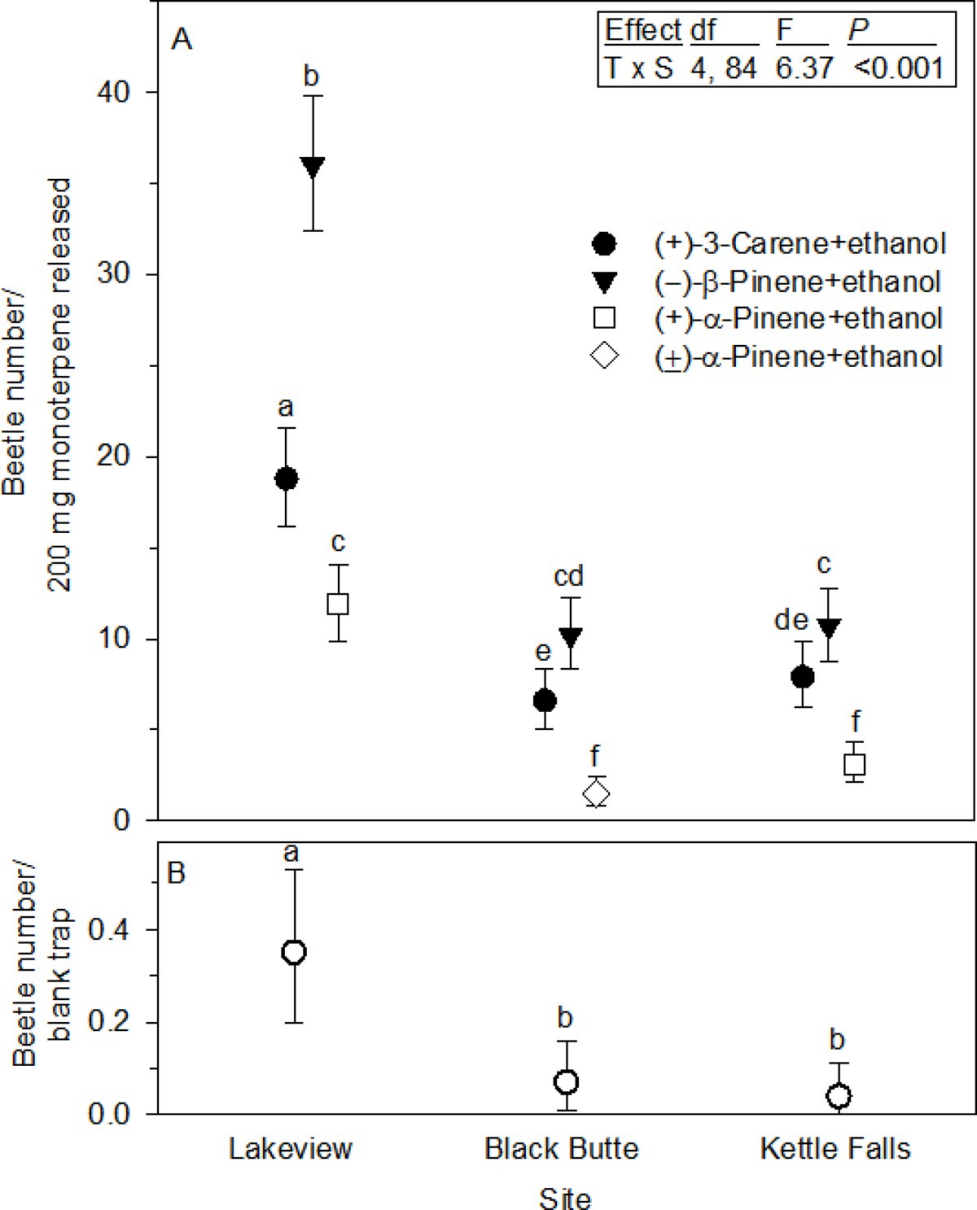
Number of *Dendroctonus valens* captured (mean ± 95% CIs). (A) Number/200 mg of monoterpene released/trap (*n* = 15) at each study site. Symbols with different letters indicate statistical differences at *P* ≤ 0.024. Inserted box shows ANOVA table results with effects abbreviated; T = terpene, S = site. (B) Number/blank trap/day; Lakeview vs. Kettle Falls (t_42_ = 4.04, *P* < 0.001), Lakeview vs. Black Butte (t_42_ = 3.40, *P* = 0.001), and no differences between Kettle Falls and Black Butte (t_42_ = 0.64, *P* = 0.526). Values in (A) and (B) are back-transformed geometric means and 95% CI.

A total of 42,941 *D*. *valens* were caught across all three sites, with just over half at Lakeview, followed by Kettle Falls and finally Black Butte (Table 1). Trap catch normalized per 200 mg monoterpene released showed a lure by site interaction (Fig 3A). Within each site, (–)-β-pinene+ethanol consistently attracted the most beetles, followed by (+)-3-carene+ethanol, and finally (+)-α-pinene+ethanol or (±)-α-pinene+ethanol the least, and they were all statistically different from one another. Blank trap beetle numbers could not be normalized by monoterpene release, so mean number/trap/day were compared by site (Fig 3B) to show they tended to track the total beetle catch by site (Table 1). Beetle catch among sites varied, as expected, because the tests were conducted during different temporal segments of the *D*. *valens* flight period, and for varying numbers of days.

**Table 1:** Total number of *Dendroctonus valens* captured (mean female proportion) at each site and each monoterpene+ethanol lure type.

	Lure type						
Site	(+)-3-Carene	(±)-α-Pinene, (+)-α-pinene	(–)-β-Pinene	Blank traps	Total catch	All traps with lures	Trap days
Black Butte	2957 (0.56)	854 (0.57)	5631 (0.54)	76 (0.45)	9518	(0.55a)	41
Kettle Falls	3253 (0.58)	1764 (0.67)	6093 (0.60)	25 (0.48)	11135	(0.62b)	29
Lakeview	5373 (0.51)	4475 (0.65)	12214 (0.53)	226 (0.47)	22288	(0.53a)	32
Total	11583 (0.55)	7093 (0.60)	23938 (0.56)	327 (0.47)	42941	(0.57)	

Notes: (±)-α-Pinene was used at Black Butte and (+)-α-pinene at Kettle Falls and Lakeview. Total catch numbers were not analyzed statistically. Female proportions in traps with lures were determined with a 10% subsamples from all beetles they captured. For blank traps, female proportions were calculated from the total catch of all blank traps within a site, because numbers were low, and proportions not possible with a single beetle. Female proportions were statistically analyzed for all traps having monoterpene lures, with ANOVA results showing a site effect difference (*P* = 0.005), a lure effect marginal difference (*P* = 0.078), and no site by lure interaction (*P* = 0.736). The ANOVA mean female proportions are presented by site in the column titled All traps with lures where those followed by different letters are statistically different at *P* ≤ 0.015, and for each lure type in the Total row with no means comparisons since they were marginally different.

Female proportions were statistically different among sites, with no interactions among lures (Table 1). Kettle Falls traps had a higher female proportion than Black Butte and Lakeview, and the latter were not different from one another, however, these could be influenced by the same site differences mentioned above for the beetle catch. Blank traps captured slightly fewer females than males, whereas traps with monoterpene lures tended to capture more females, with differences among lure types marginally significant. The trend was a higher proportion of females responding to (+)-α-pinene lures than to (+)-3-carene and (–)-β-pinene lures.

### Field monoterpene and ethanol release

**Fig 4:**
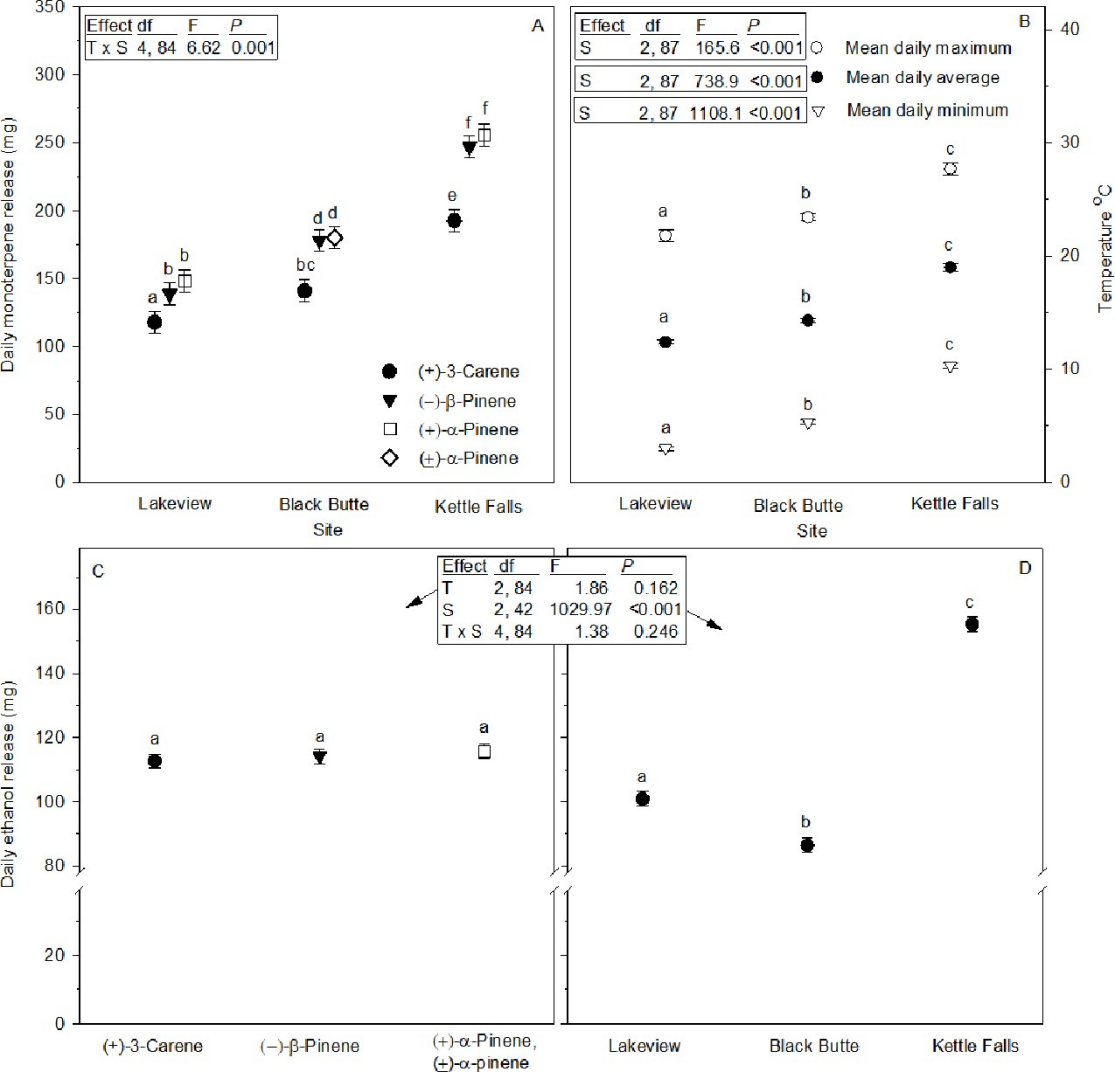
Field release rates and temperatures of monoterpene and ethanol lures (means ± 95% CIs). (A) Daily monoterpene release rates at each study site (*n* = 15). (+)-α-pinene, (±)-α-pinene, or (–)-β-pinene had higher releases because three bottles of each were needed to obtain similar release by one bottle of (+)-3-carene based on laboratory results, Fig 5C and 5D. (B) Daily maximum, minimum and average temperatures in lure mixing jars at each site. (C) Daily ethanol release rates for each monoterpene lure type across all three study sites (*n* = 45). (D) Daily ethanol release rates within each site across all monoterpene lures (*n* = 45). Symbols with different letters in A, C, and D are statistically different at *P* ≤ 0.05 for all comparisons, and at *P* ≤ 0.001 for all B comparisons. Inserted boxes show ANOVA table results for each statistical analysis with effects abbreviated; T = terpene, S = site. Note Y-axis breaks in graph C and D.

Daily monoterpene field release rates depended on monoterpene type and site, as indicated by the interaction (Fig 4A). At each site, the (+)-3-carene release was statistically lower than (±)-α-pinene or (+)-α-pinene, and (–)-β-pinene, with no differences between the pinenes. Highest release rates were at Kettle Falls, where (+)-α-pinene, (–)-β-pinene, (+)-3-carene were 1.7, 1.8 and 1.6 times greater than at Lakeview because of differences in their temperatures as described below.

Ethanol pouch release rates varied by site, but not among monoterpene types, and there was no site by lure interaction (Fig 4C and 4D). Ethanol release was highest at Kettle Falls, followed by Lakeview, and finally Black Butte because its pouches had restricted surface areas; all three were statistically different.

### Field lure temperatures

At Black Butte, two ibutton data loggers were attached inside mixing jars, on opposite walls to assess within jar variation, since each jar was randomly positioned relative to potential shade influences from the adjacent trap and surrounding tree overstory. The three lure types experienced no differences for mean daily average, maximum, or minimum temperatures, and within mixing jar temperature variation was relatively small (see S4 Appendix for details). At Black Butte, Kettle Falls and Lakeview, the mean daily average, maximum, and minimum temperatures were statistically different among sites (Fig 4B). Kettle Falls had the highest temperatures, followed by Black Butte, and Lakeview the lowest.

### Laboratory monoterpene evaporation and LDPE release

**Fig 5:**
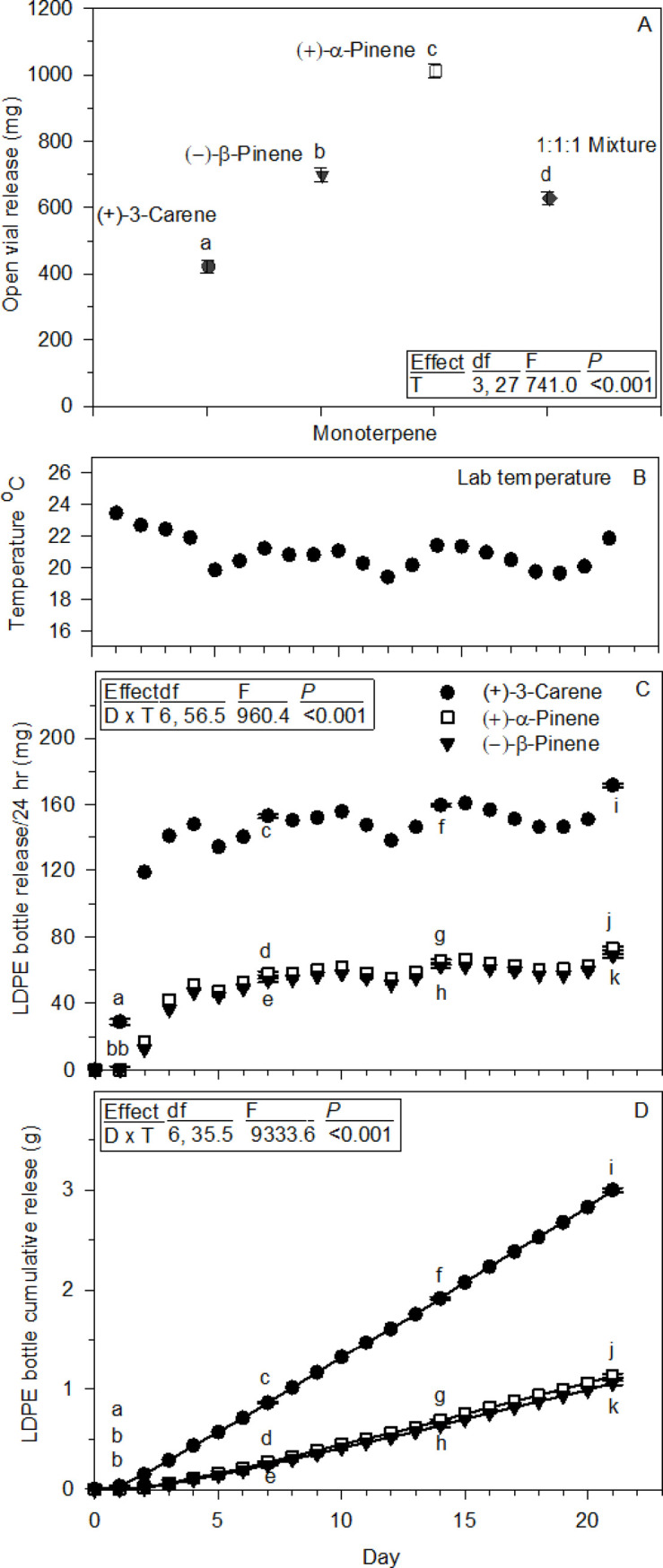
Monoterpene evaporation and LDPE release rates in the laboratory (means ± 95% CIs). (A) Evaporation of (+)-α-pinene, (–)-β-pinene, (+)-3-carene, and their 1:1:1 mixture from vials open 17 days (*n* = 10). (B) Laboratory mean daily average temperature for LDPE release of individual monoterpenes in C and D. (C) Each monoterpenes 24 h release rate from LDPE bottles over 21 days (*n* = 10). (D) Each monoterpenes cumulative release from the LDPE bottles in C. In A, C, and D the symbols with different adjacent letters are statistically different from one another at *P* < 0.001 for all comparison. Inserted boxes show ANOVA tables for each statistical analysis with effects abbreviated; D = day, T = terpene.

Open vial evaporation rates were lowest for (+)-3-carene, then (–)-β-pinene, and finally (+)-α-pinene the highest, with an intermediate rate for the 1:1:1 mixture being more similar to (–)-β-pinene than the other two, and all were statistically different (Fig 5A). The (+)-α-pinene rate was 2.4 times greater than (+)-3-carene, and 1.4 times greater than (−)-β-pinene. Mean daily average hood temperature was 21.3°C (± 2.2 SD), range 19.1 to 25.1°C.

LDPE bottle 24 h release of the individual monoterpenes increased rapidly until day 4, then leveled off and started tracking laboratory temperature changes, with a day and monoterpene interaction (Fig 5B and 5C). (+)-3-Carene 24 h rates were statistically higher than (+)-α-pinene and (–)-β-pinene at day 1, 7, 14 and 21. On day 1 it was 48.2 times higher than both (+)-α-pinene and (–)-β-pinene, and by day 14 just 2.4 and 2.6 times higher, respectively. The (+)-α-pinene 24 h release rate was statistically higher than (–)-β-pinene on days 7, 14, and 21. Their cumulative releases from the same experiment provides another perspective, and was also dependent on a day by monoterpene interaction (Fig 5D). At day 1, 7, 14 and 21, all were statistically different from one another, except for day 1 (+)-α-pinene and (–)-β-pinene. Cumulative (+)-3-carene release on day 14 was 2.8 and 3.0 times higher than (+)-α-pinene and (–)-β-pinene. Mean daily average hood temperature was 20.9°C (± 1.1 SD), range 19.4 to 23.4°C (Fig 5B).

**Fig 6:**
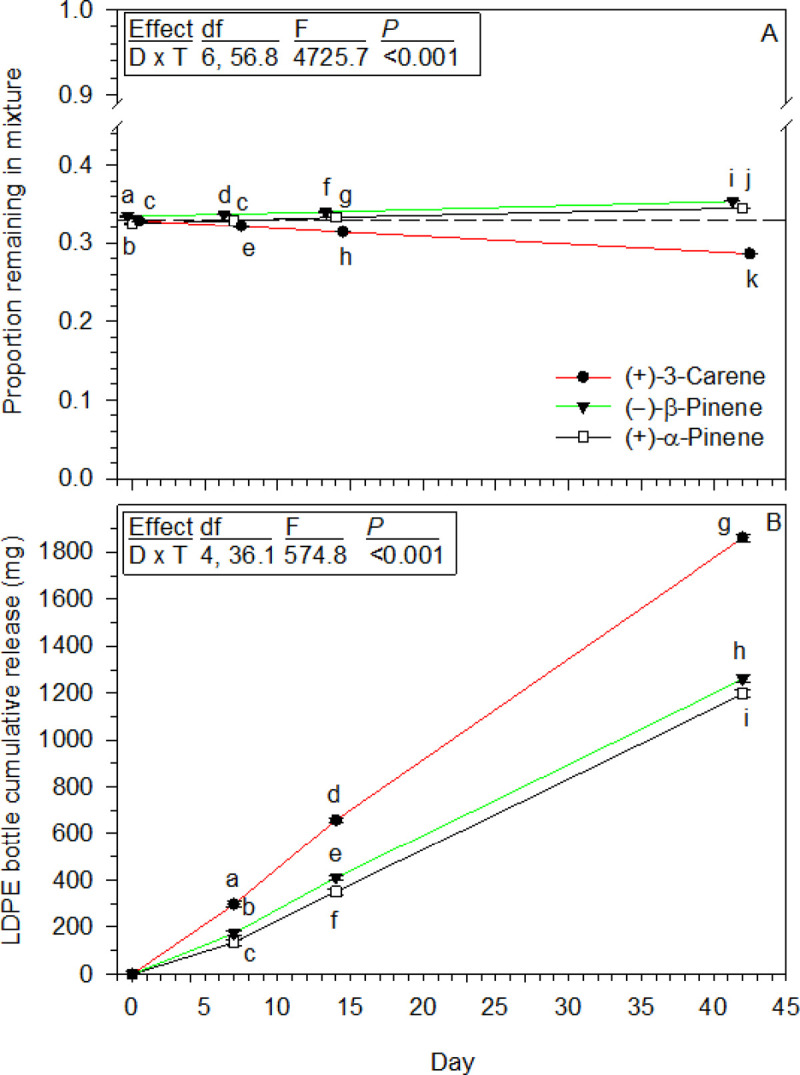
LDPE bottle release of a 1:1:1, (+)-α-pinene:(−)-β-pinene:(+)-3-carene, mixture in the laboratory (means ± 95% CIs). (A) Proportions of each monoterpene remaining in the mixture at 0, 7, 14, and 42 days (*n* = 10). The dashed line is a reference = 0.333 proportion to help illustrate differences. (B) Cumulative release of each monoterpene at the same four days as A. In A and B the symbols with different letters are statistically different from one another at *P* < 0.001 for all comparisons. Note Y-axis break in graph A. Inserted boxes show ANOVA tables for each statistical analysis with effects abbreviated; D = Day, T = terpene.

LDPE bottle release of a 1:1:1 [(+)-α-pinene:(–)-β-pinene:(+)-3-carene] mixture was monitored in two laboratory experiments, the first lasting 42 days, and the second for 21 days along with release of 1:1:3 and 1:4:1 mixtures used as lures by others [6]. The first experiment shows (+)-3-carene declining in proportion while the two pinenes slowly increased over time (Fig 6A), but their cumulative releases are visually more dramatic (Fig 6B). Both analyses revealed a day by monoterpene interaction, with all comparisons among the three at each day statistically different in both data sets (Fig 6A and 6B). At day 42 in the first experiment, (+)-3-carene cumulative loss was 1.6 times higher than (+)-α-pinene and 1.5 times higher than (–)-β-pinene, while the latter was only 1.1 times greater than (+)-α-pinene (Fig 6B). Mean daily average hood temperature was 23.6°C (± 1.3, SD), range 18.5 to 29.3°C.

**Fig 7:**
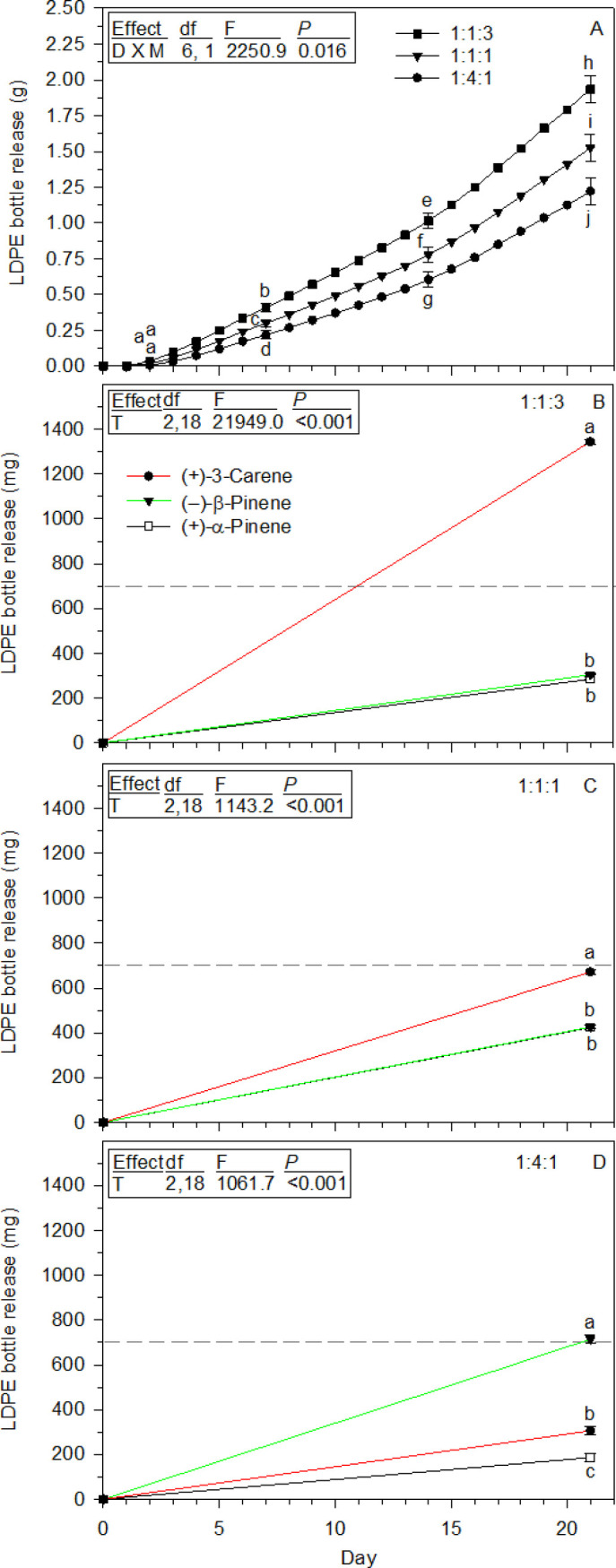
LDPE bottle cumulative release of (+)-α-pinene:(–)-β-pinene:(+)-3-carene mixtures and each individual monoterpene after 21 days in the laboratory (means± 95% CIs, *n* = 10). (A) Cumulative release of each mixture. (B) 1:1:3 Mixture, individual monoterpene release. (C) 1:1:1 Mixture, individual monoterpene release. (D) 1:4:1 Mixture, individual monoterpene release. Symbols with different letters are statistically different from one another with *P* < 0.009 for all comparisons in A, and *P* < 0.001 for all comparisons in B-D. Inserted boxes show ANOVA tables for each statistical analysis with effects abbreviated; D = Day, M = Mixture, T = Terpene. The dashed line at 700 mg is a reference to help illustrate differences.

In the second experiment, the releases of 1:1:1, 1:1:3, and 1:4:1 mixtures were compared (Fig 7). The 1:1:1 mixtures cumulative release patterns for the three monoterpenes were similar to the first experiment (Figs 7C and 6B,) with (+)-3-carene 1.6 times higher than both (+)-α-pinene and (–)-β-pinene, and the latter two not different statistically at day 21. The 1:1:3 solution initially contained 3 times more (+)-3-carene than (+)-α-pinene and (–)-β-pinene, yet the (+)-3-carene cumulative release at 21 days was 4.4 and 4.5 times higher, respectively, with no statistical difference for the pinenes (Fig 7B). The 1:4:1 mixture contained 4 times more (–)-β-pinene than (+)-3-carene, but its release was only 2.3 times higher than (+)-3-carene at 21 days (Fig 7D). Also, the 1:4:1 mixture had equal proportions of (+)-α-pinene and (+)-3-carene, yet 1.6 times more (+)-3-carene was released. Mean daily average hood temperature was 20.8°C (± 1.5, SD), range 18.3 to 23.4°C.

Differences in (+)-α-pinene and (–)-β-pinene releases from LDPE bottle with a 1:1:1 mixture were calculated for day 2 and compared to their day 2 releases from separate bottles. Their 1:1:1 mixture values were estimated from quantities released through day 7 (Fig 6B) and back calculated to day 2 as a linear change observed for the total mixture (Fig 7A). This indicated (+)-α-pinene and (–)-β-pinene contributed 22.5 and 28.8%, respectively, to the total 1:1:1 mixture released. When bottles released each separately (Fig 5D) they represented 9.6 and 7.4%, respectively of the total amount for the three compounds summed together at day 2. Thus, they were released 2.3 and 3.9 times faster, respectively, from the 1:1:1 mixture. All laboratory experiments demonstrate (+)-3-carene is absorbed and moves more rapidly through LDPE bottles than both (+)-α-pinene and (–)-β-pinene, and when combined in a mixture such as 1:1:1, the presence of (+)-3-carene increases absorption and release of both pinenes.

## Discussion

### Primary attraction to (−)-β-pinene+ethanol

(−)-β-Pinene+ethanol was a stronger primary attractant for *D*. *valens* than (+)-3-carene+ethanol at all three sites in Washington and Oregon, whereas (+)-α-pinene+ethanol, or racemic (±)-α-pinene+ethanol attracted the fewest beetles. Whether (−)-β-pinene without ethanol is a stronger primary attractant than (+)-3-carene in these forests remains to be determined. If so, the Pacific Northwest beetle responses would be distinctly different from *D*. *valens* across California at multiple locations, Wisconsin, Pennsylvania and China where (+)-3-carene > (−)-β-pinene ≥ (+)-α-pinene is the consistent order of attraction strength [6, 7], with only one exception in California where (−)-β-pinene was the strongest attractant [5], but this has not been repeated in the more recent tests above.

The *D*. *valens* responses here do not likely result from genetically isolated beetle populations, or their adaptation to high β-pinene concentrations in northwest hosts, or unusual lure release rates. Washington and Oregon *D*. *valens* are genetically similar to one another, and to those in Idaho, Montana and northern half of California [40]. They cluster together into a western population based on microsatellite markers, and are distinct from three other isolated populations in North America. Adaptation to host pine with high β-pinene concentrations is probably not involved based on two observations. First, (+)-3-carene is the dominant monoterpene in northwest ponderosa pine; at Lakeview and Kettle Falls the phloem oleoresin averaged 46 and 49% 3-carene, then 14.7 and 15% β-pinene, with 7.7 and 8.3 α-pinene, respectively (S4 Appendix), similar to the xylem resin reported by Smith in the same geographic areas [43]. Second, California ponderosa pine have high β-pinene, often the second most abundant or dominant compound at southwestern locations [43], and yet (+)-3-carene attracts more beetles across multiple California sites [7]. Also, there was nothing unusual regarding our monoterpene release rates, as they were within or near ranges used in other studies [6, 7]. Finally, the beetle’s response to (−)-β-pinene+ ethanol in Washington and Oregon could be synergistic as it attracted 1.4 to 1.9 times more beetles than (+)-3-carene+ethanol, a lure known to cause a synergistic beetle response [53], but additional trials are needed to confirm this behavior.

The *D*. *valens* responses among Black Butte lures were similar to Lakeview and Kettle Falls, even though Black Butte lures had differences worth mentioning. First, the ethanol release rates were lower because of reduced pouch surface area. Without this restriction, ethanol release would have reached levels between Lakeview and Kettle Falls, based on monoterpene release patterns and site temperatures, and more ethanol would have increased the beetle catch for all Black Butte lures given its strong influence on pioneering *D*. *valens* when mixed with monoterpenes [20, 48, 58]. Black Butte also had racemic (±)-α-pinene lures, rather than the 97% (+)-α-pinene at the other two sites. Previous studies with both isomers have shown mixed beetle responses. At one California site (+)-α-pinene was a much stronger attractant than (−)-α-pinene, and the latter was considered an interruptant [5]. In another study (+)-α-pinene attracted more beetles in northern California, Wisconsin, and Pennsylvania, but similar numbers responded to each isomer in central and southern California, Mexico, and China [6, 7]. When ethanol is present, the ratio of ethanol:α-pinene influences the isomer having stronger attraction [63]. At 5:1, ethanol:(+)-α-pinene attracted 44% of the beetles responding to ethanol:(−)-α-pinene, and at 1:1 the opposite occurred, ethanol:(−)-α-pinene attracted 41% as many beetles as ethanol:(+)-α-pinene. Here, the *D*. *valens* responses among lures at Black Butte with (±)-α-pinene+ethanol were similar to Kettle Falls with (+)-α-pinene+ ethanol. This combined with previous results imply minimal differences between the beetle’s attraction to (±)-α-pinene+ethanol and (+)-α-pinene+ethanol. Finally, while beetle responses among lures at Black Butte and Kettle Falls were similar, at Lakeview there was a much larger response to (–)-β-pinene+ethanol resulting in a significant site-by-lure interaction. The Lakeview response was not caused by unusual monoterpene release rates, because they were more similar to one another than among lures within the other two sites. The higher beetle density at Lakeview may have been a factor.

Prineville 1:1:1+ethanol attracted 1.5 times more beetles than 1:1:1 (S2 Appendix), similar to a California study where the former lures attracted 1.2 times more beetles than the latter [56], with no statistical difference at either site. A much higher response to 1:1:1+ethanol lures would seem likely since (+)-3-carene+ethanol attracted two times more beetles than (+)-3-carene [53], and here the (–)-β-pinene+ethanol attracted 1.4 to 1.9 times more beetles than (+)-3-carene+ethanol. Ethanol does not enhance detection of 1:1:1 mixtures to the same extent as each individual monoterpene, possibly from interference among one another in a mixture as suggested for α-pinene isomers [5].

All monoterpene and ethanol release rates (excluding Black Butte ethanol lures with reduced surface area) were mitigated by their deployment dates and duration on site, but still strongly tracked with site temperatures related to their elevations. Lakeview lures at 1725 m had the lowest release rates and temperatures, while Kettle Falls at 510 m had the highest release rates and temperature, but also was deployed later in the spring when temperatures were warmer.

### Monoterpene structures, physicochemical properties and LDPE release

Beetle detection and primary attraction are linked to the monoterpenes structural compositions and associated physicochemical properties. All three tested are bicyclic C_10_H_16_ isomers with one six-carbon ring and one double bond (Fig 2). The two pinenes are structurally most similar with a second four-carbon ring involving a quaternary carbon with two methyl groups bridged across the cyclic six carbons, and one endocyclic or exocyclic double bond being their only difference. The second ring in (+)-3-carene is the major structural feature setting it apart from the two pinenes, it has only three carbons and is positioned at one edge of the six-carbon ring, opposite the endocyclic double bond (Fig 2). Each structure effects the strength of intermolecular forces among their interacting molecules, and the force strengths are inversely related to each compounds vapor pressure [64, 65] and associated liquid evaporation rate starting with the lowest for (+)-3-carene < (–)-β-pinene < (+)-α-pinene. Lures releasing them by direct evaporation require adjustments to provide similar release rates, such as using different numbers of containers, or modifying sizes of container openings, etc. Their release rates also change dramatically when dispensed through LDPE bottles.

(+)-3-Carene had the fastest LDPE bottle release, but slowest liquid evaporation of the three monoterpenes. The two pinenes have distinct evaporation rates, but nearly the same LDPE bottle releases, and substantially slower than (+)-3-carene. It took four days after filling LDPE bottles for the 24 h release rates to stabilize, with (+)-3-carene remaining between 2–3 times faster than both pinenes through day 21. This indicated their field releases would be stable for extended periods, but multiple bottles of both pinenes were needed to provide quantities similar to one bottle of (+)-3-carene. (+)-3-Carene in a 1:1:1 mixture also released more rapidly than the two pinenes from laboratory LDPE bottles, and at day 2 (+)-α-pinene and (–)-β-pinene releases were 2.3 and 3.9 times faster than their individual releases from the bottles. It is also notable, each monoterpenes release from all mixtures was not directly related to their proportions in the solutions.

Insight into factors influencing the three monoterpenes LDPE sorbtion, movement, and release comes from detailed studies of interactions between organic compounds and polymeric materials used for food packaging, including LDPE [66–71]. First, are the LDPE parameters including its degree of crystallinity, size-shape of internal void spaces, and polymer polarity, among others [66–69, 71]. The first two are derived from its polymer chain structure with an abundance of short and long length side branches creating various levels of crystallinity where highly organized chains limit accessibility [67, 68, 70, 71], but interspersed are amorphous areas where less organized chains have internal void spaces, or free volume, for molecules to penetrate and diffuse [68, 69, 71]. Second, is the organic compounds characteristics including molecular weight (here all the same), polarity, and flexibility determined by dimensions and shapes of internal atomic groups [66, 69, 71]. A relevant monoterpene example is the distinct behaviors of limonene, myrcene, and linalool, listed from high to low adsorption and diffusion coefficients in LDPE film [71]. Their modes of diffusion were linked to rigid (rings) and flexible groups of atoms within their structures, as shown for other compounds [66, 71]. Thus, the disparate (+)-3-carene LDPE release is likely a function of the three-carbon ring size and rigidity, plus the impact of its edge attachment on flexibility of atom groups in the six-carbon ring, as these are the major structural features distinguishing it from the two pinenes with their analogous ring structures and release rates. A third factor is change in polymer configuration from organic compound interactions, and includes swelling with increases in free volume allowing more rapid monoterpene movement over time [67, 72], as observed for all three here, but especially (+)-3-carene. With a 1:1:1 mixed solution, rapid (+)-3-carene uptake and LDPE interactions likely caused free volume swelling and improved access for (+)-α-pinene and (–)-β-pinene, allowing them faster release than when dispensed separately from LDPE bottles.

### Ecological role of β-pinene+ethanol attraction

The ecological function and importance of *D*. *valens* attraction to (–)-β-pinene+ethanol over (+)-3-carene+ethanol in northwest forests remains unclear since pine oleoresins are typically complex mixtures [43, 44], and a 1:1:1 mixture seems to interfere with ethanol’s ability to increase its attraction strength. Across much of the native ponderosa pine range, (+)-3-carene is either the dominant, or co-dominant component, followed by β-pinene or myrcene [43, 44]. Nevertheless, tissue ethanol concentrations in fire damaged ponderosa pine strongly influenced the initial trees selected and attacked by pioneering *D*. *valens*, and also their gallery entrance locations on the boles of attacked trees [20]. Complete monoterpene profiles for these burned trees were not analyzed, only α-pinene, but they were located in the general area sampled by Smith [43] (29 and 54 km south of the two fires we sampled) with (+)-3carene, β-pinene, and α-pinene at 61.7, 15.8, and 5.0%, respectively. These (+)-3-carene proportion are similar to the 60% in 1:1:3 [(+)-α-pinene:(–)-β-pinene:(+)-3-carene] mixed lures tested in China that attracted nearly 2 times more *D*. *valens* than the 1:1:1 lures with 33.3% (+)-3-carene [6]. How the presence of ethanol would impact the 1:1:3 attraction, or other mixture combinations remains to be determined. Ethanol release from stress-induced ponderosa pine in southwestern California, where β-pinene is the dominant compound, would likely enhance substantially their probability of detection by pioneering *D*. *valens*.

### Gender responses

Gender response differences among the three monoterpene lures were minimal, although a higher proportion of females were attracted to (+)-α-pinene+ethanol than the other two lure types, with a marginal statistical difference, in part because of their lower response to (±)-α-pinene lures at Black Butte. Here again, the ecological importance may be limited, unless it also occurs outside northwest forests, then it might influence gender responses for those pine species, or at geographic sites where α-pinene dominates the oleoresin, as in Arizona ponderosa pine [43]. However, this has not been observed in China; both sexes respond the same to (+)-α-pinene, (–)-β-pinene, (+)-3-carene, or their various mixtures [6], and show no gender-related difference in attraction to *P*. *tabuliformis* tree size, volatiles collected from their bark, or synthetic blends mimicking the oleoresin compositions containing primarily α-pinene, or both pinenes, when tested in olfactometers [45].

## Conclusion

(–)-β-Pinene+ethanol is the strongest kairomone lure for *D*. *valens* tested so far in Pacific Northwest forests, with utility for research projects, or monitoring programs interested in detecting them. It would be particularly useful in this region for trapping at ports of entry receiving international cargo from China to detect potential reintroductions of *D*. *valens* carrying foreign symbiotic microbes that may include new pathogens and/or more virulent pathogen strains than those currently present in North American pine forests. Molecular diagnostics of the microbial symbionts would be required for pathogen detection and verification. While the probability of such an introduction is low, should it occur, there exists a continuous and extensive supply of fire injured ponderosa pine, as well as other pine species for these invasive beetles to select as hosts across North American forests. If any new pathogens were to establish, their rapid spread through burned pine communities by *D*. *valens* seems highly feasible.

## Supporting information

S1 Appendix Detailed study site descriptions for Black Butte, Kettle Falls, Lakeview and Prineville. (DOCX)

S2 Appendix Prineville trapping experiment comparing 1:1:1 and 1:1:1+ethanol attraction of *D. valens*. (DOCX)

S3 Appendix Phloem constitutive monoterpenes in ponderosa pine at Lakeview and Kettle Falls. (DOCX)

S4 Appendix Lure temperatures in mixing jars at Black Butte. (DOCX)
