# Supplementary material for: Red turpentine beetle primary attraction to (–)-β-pinene+ethanol in US Pacific Northwest ponderosa pine forests
Source: PLoS One. 2020 Jul 30;15(7):e0236276. doi: 10.1371/journal.pone.0236276 (PMC7392304; doi:10.1371/journal.pone.0236276)
Supplement: S1 Appendix — (DOCX) [file pone.0236276.s001.docx]

**S1 Appendix. Detailed study site descriptions for Black Butte, Kettle Falls, Lakeview and Prineville**

Black Butte (44°21’05”N, 121°38’20”W, elevation 1015 m) is on the Deschutes National Forest, Sisters Ranger District (RD), approximately 9 km aerial distance northwest of Sisters, Oregon (OR) and was prescribed burned 30 April, 2015. The site is flat, clear cut in the 1930’s, and thinned from below in 2011. It is currently stocked with 32.8 m^2^/ha basal area of ponderosa pine with a mean stem diameter of 52.8 cm ranging from 2.5 to 94.7 cm. Dominant tree mean height is 27.1 m. Understory shrubs are snowbrush ceanothus (*Ceanothus velutinus* Dougl. ex Hook.), and greenleaf manzanita (*Arctostaphylos patula* Greene). These stand summary metrics were obtained from measurements by Deschutes National Forest, Sisters Ranger District silviculture personnel.

Kettle Falls (48°35’21”N, 118°08’45”W, elevation 510 m) is on the Sherman Creek Wildlife Area, Washington (WA) State Department of Fish and Wildlife, located about 5 km aerial distance southwest of Kettle Falls, WA, and was prescribed burned in several small sub-units between 21 and 29 June, 2016. The site has gentle 0-15% easterly slopes and is sparsely stocked with 12.09 m^2^/ha basal area of ponderosa pine with a mean stem diameter of 54.1 cm, ranging from 26.2 to 85.3 cm. Dominant tree mean height and age was 28.3 m, and 85.3 years, respectively. Dominant understory shrubs are choke cherry (*Prunus virginiana* L.) and bitterbrush [*Purshia tridentata* (Pursh) DC]. During the 2017 trapping period additional prescribed burns were conducted in adjacent stands, one burned two days before and another the day of the first beetle collection, likely influencing the number of beetles captured during the second trap period. Kettle Falls summary metrics were from 30 systematically established variable radius plots overlapping the trap positions, using a 10 basal area factor prism. Species, diameter and height of all trees within these plots were measured, and the largest diameter tree of each species was cored to the pith with a 5 mm diameter increment borer; growth rings were counted in the field to estimate age.

Lakeview (42°05’24”N, 120°51’39”W, elevation 1725 m) is on the Fremont-Winema National Forest, Lakeview RD, approximately 43 km aerial distance southwest of Lakeview, OR and prescribed burned 11 to 13 May, 2016. Aspects are variable with slopes from 0 to 35%. This mixed conifer site is stocked with 12.5 m^2^/ha basal area of ponderosa pine, with a mean stem diameter of 59.9 cm ranging from 6.6 to 111.0 cm; 1.8 m^2^/ha basal area of incense cedar [*Calocedrus decurrensv* (Torr.) Florin] with a mean stem diameter of 54.4 cm ranging from 8.4 to 102.6 cm; and 1.08 m^2^/ha basal area of white fir [*Abies concolor* (Gordon & Glend.) Lindl. ex Hildebr] with a mean stem diameter of 45.2 cm, ranging from 35.6 to 55.4 cm. Dominant tree mean height and age is 28.6 m and 171 years, 22.7 m and 133 years, and 19.2 m and 115 years, for ponderosa pine, incense cedar, and white fir, respectively. Primary understory shrubs are snowbrush ceanothus (*Ceanothus velutinus*), Saskatoon serviceberry [*Amelancher alnifolia* (Nutt.) Nutt. ex M. Roem.], and greenleaf manzanita (*Arctostaphylos patula*). On the first beetle collection date there was a prescribed burn in a stand about 3.5 km aerial distance from our site, but it appeared to be low intensity, and likely had minimal influence on the number of beetles captured during the second trap period. Lakeview stand summaries were from 15 systematically established variable radius plots as described above for Kettle Falls.

Prineville (44°25'15"N; 120°25'40"W, elevation 1450 m) is on the Ochoco National Forest, Prineville RD, approximately 37 km aerial distance northeast of Prineville, OR on the prescribed burned named Canyon 66 of about 2023 ha, ignited 3 and 4 September, 2019. Traps were located in burned areas where ponderosa pine was the most abundant conifer and fire injury was sufficient to attract *D. valens*, with some trees already attacked. Traps were positioned at variable aspects and slopes from 0 to 35% or more. Twenty-four traps were located over an area non-commercially thinned in 2015, leaving a basal area of approximately 13.76 m^2^/ha, with stem diameters ranging from 10.2 to 40.6 cm, with an average of about 22.9 cm. The remaining six traps were in an area not mechanically treated recently. It has a basal area of about 27.5 m^2^/ha with stem diameters from 2.5 to 61.0 cm, and average diameter of 20.3 cm. The dominant shrub is common snowberry [*Symphoricarpos albus* (L.) S.F. Blake], with pinegrass (*Calamagrostis rubescens* Buckley) and elk sedge (*Carex geyeri* Boott) the dominant ground cover. Prineville stand information was provided by the Ochoco National Forest, Prineville Ranger District Silviculturist.
