# Supplementary material for: Red turpentine beetle primary attraction to (–)-β-pinene+ethanol in US Pacific Northwest ponderosa pine forests
Source: PLoS One. 2020 Jul 30;15(7):e0236276. doi: 10.1371/journal.pone.0236276 (PMC7392304; doi:10.1371/journal.pone.0236276)
Supplement: S2 Appendix — (DOCX) [file pone.0236276.s002.docx]

**S2 Appendix. Prineville trapping experiment comparing 1:1:1 and 1:1:1+ethanol attraction of *D. valens***

This experiment near Prineville was conducted to further evaluate the limited increase in *D. valens* attraction to traps baited with 1:1:1+ethanol lures, compared to those baited with 1:1:1 lures, as observed by Fettig et al. [56]. The test site (44°25'15"N; 120°25'40"W, elevation 1450 m) is on the Ochoco National Forest, Prineville Ranger District, approximately 37 km aerial distance northeast of Prineville, OR (see S1 Appendix for further details).

Lures

Prineville lures were prepared with the same LDPE bottles, monoterpenes and ethanol used at Kettle Falls and Lakeview. A 1:1:1 (+)-α-pinene (97%):(−)-β-pinene (≥ 97%):(+)-3-carene (≥ 90%) mixture was added to LDPE bottles and combined with ethanol pouches in a large high density polyethylene mixing jar (9.3 W x 16.8 L cm cap on, 946 ml volume; Uline). Five equally spaced ventilation holes were drilled on the bottom (1. 9 cm) in an X pattern. A 7.6 cm diameter opening was cut in the cap and fitted with a wire mesh screen (1.5 x 1.5 mm mesh openings) to prevent access to beetles and small mammals. The outside jar wall was wrapped with a layer of aluminum foil to reduce solar heating. Each jar received three LDPE bottles, or three bottles+four ethanol pouches. One pair of pouches was wrapped around the inside wall at the jars bottom, and another pair on top, with an opening in the center where the LDPE bottles were placed, just beneath the cap screen. LDPE bottles were weighed before and after the test as described for Kettle Falls and Lakeview to determine the monoterpene release. Ethanol pouches were not weighed. Mixing jars were attached to traps outside the 6^th^ funnel above the collection cup.

Traps and Field Test

Thirty, 16 unit funnel traps were installed in a randomized complete block design with 15 blocks and two randomly assigned lures with a 1:1:1 mixture, or 1:1:1 mixture+ethanol, within blocks. Lures were attached on 18 September and beetles collected on 11 October, 2019, 23 days total. The total number of *D. valens* caught in each trap was summed over the entire period without normalizing to the monoterpene release rates as there was no difference between lure types. Beetle catch was analyzed as a randomized complete block mixed-model ANOVA design with two lure types. Block was modeled as a random effect, and lure type as a fixed effect.

Field Lure Temperatures

Temperatures inside each Prineville mixing jar were recorded with an ibutton temperature data logger attached to the cap preventing them from falling out a bottom hole in the mixing jar. They were not analyzed statistically.

**Results**

Prineville beetle numbers were low, as expected for fall trapping, but sufficient to recognize a response difference between lures, if present. Traps with 1:1:1+ethanol lures caught a mean of 3.1 beetles/trap (1.7, 4.6; 95% CI), whereas those with 1:1:1 lures caught 2.1/trap (0.6, 3.5; 95% CI), and they were not statistically different (F_1, 14_ = 1.38, *P* = 0.259). The mean total monoterpene release per trap for 1:1:1 lures was 2.666 g (2.484, 2.848 g, 95% CI), and 2.696 g (2.515, 2.878 g, 95% CI) for 1:1:1+ethanol lures; nearly identical (F_1,14_ = 0.15, *P* = 0.706). Lures experienced especially low temperatures with mean daily average, maximum, and minimum at 6.2^o^C (± 0.7 SD), 14.7°C (± 3.3), and 1.6°C (±0.6), respectively. Eighteen of the 23 days had maximum temperatures above 10^o^C.
