# Supplementary material for: Red turpentine beetle primary attraction to (–)-β-pinene+ethanol in US Pacific Northwest ponderosa pine forests
Source: PLoS One. 2020 Jul 30;15(7):e0236276. doi: 10.1371/journal.pone.0236276 (PMC7392304; doi:10.1371/journal.pone.0236276)
Supplement: S3 Appendix — (DOCX) [file pone.0236276.s003.docx]

**S3 Appendix. Phloem constitutive monoterpenes in ponderosa pine at Lakeview and Kettle Falls**

**Materials and methods**

Samples of phloem were collected from ten ponderosa pine located just outside the burn perimeter at Lakeview on 21 September, 2016 and Kettle Falls on 14 June, 2017. Trees with diameters comparable to the live, dominant trees in the burn were selected as encountered. One tissue core was removed with an increment borer (5 mm dia.) about 15 cm above the forest floor with no regard to the cardinal direction of the stem side selected. The phloem was sealed in a 4 ml screw cap vial after removing outer bark and xylem, then immediately frozen with dry ice and transported to the laboratory for storage in a -36°C freezer until processed for gas chromatography (GC) analysis.

Prior to extraction the phloem samples were thawed to room temperature and weighed into 4 ml vials, then covered with hexanes (Fisher Scientific, HPLC grade, Fair Lawn, NJ, USA). Samples weighing ≥ 45 mg fresh mass received 500 µl of solvent and those less than 45 mg received 250 µl. Vials were placed on an orbital shaker at 70 rpm for 24 h, then the phloem was removed and extracts held at 5°C until analyzed by GC.

All GC analyses were performed with the same instrument, column, carrier gas, and injector and detector temperatures described in the main manuscript for lure mixtures, except the split was set at 1:10. The oven program for Lakeview phloem started at 60°C then increased 3°C/min up to 300°C, with no final hold. For Kettle Falls samples the oven program was modified slightly, it started at 50°C with a 10 min hold, then 3°C/min up to 180°C, with no final hold. Two µl of each phloem extract was analyzed. All compounds were identified from retention times of authentic standards.

**Results**

Phloem tissue from trees at Lakeview and Kettle Falls had nearly the same mean proportions of constitutive 3-carene, β-pinene, and α-pinene (Table S3). Although not compared statistically, their overlapping standard errors indicate they would not be different. The mean DBH for sampled trees was 56.2 cm (±14.9 SD) at Lakeview and 53.2 cm (±13.0 SD) at Kettle Falls. They were similar in size to the mean DBH of dominant trees remaining in the adjacent prescribed burns.

**Table S3.** Percent composition of constitutive monoterpenes in phloem of ponderosa pine at Lakeview and Kettle Falls.

|  | | Lakeview | |  | | | Kettle Falls | | | |
| --- | --- | --- | --- | --- | --- | --- | --- | --- | --- | --- |
| Compounds | | Percent | Range | |  | | | Percent | | Range |
| α-Pinene | 8.3 (± 1.0) | | 3.9-14.7 | | |  | | | 7.7 (± 1.8) | 2.9-22.3 |
| Sabinene | 0.8 (± 0.1) | | 0.5-1.3 | | |  | | | 1.3 (± 0.1) | 0.8-1.7 |
| β-Pinene | 14.6 (± 3.7) | | 0.6-32.7 | | |  | | | 15.0 (± 3.9) | 0.5-33.3 |
| Myrcene | 14.9 (± 1.8) | | 8.3-24.6 | | |  | | | 11.2 (± 1.6) | 4.4-19.6 |
| 3-Carene | 46.2 (± 3.7) | | 29.2-65.6 | | |  | | | 48.5 (± 3.1) | 28.8-58.8 |
| Limonene | 11.0 (± 2.6) | | 1.5-25.7 | | |  | | | 10.6 (± 1.8) | 2.1-23.4 |
| Terpinolene | 3.5 (± 0.5) | | 1.7-6.8 | | |  | | | 4.8 (± 0.5) | 3.3-7.9 |

Notes: Values presented are means ± SE.
