# Supplementary material for: Red turpentine beetle primary attraction to (–)-β-pinene+ethanol in US Pacific Northwest ponderosa pine forests
Source: PLoS One. 2020 Jul 30;15(7):e0236276. doi: 10.1371/journal.pone.0236276 (PMC7392304; doi:10.1371/journal.pone.0236276)
Supplement: S4 Appendix — (DOCX) [file pone.0236276.s004.docx]

**S4 Appendix. Lure temperatures in mixing jars at Black Butte**

At Black Butte two ibutton data loggers were attached inside mixing jars, on opposite walls to assess within-jar variation, since each jar was randomly positioned relative to potential shade influences from the adjacent trap and surrounding tree overstory. Within-jar temperature variation was relatively small with differences of 0.3°C (± 0.3 SD), 0.7°C (± 0.6), 0.2°C (± 0.1) for means of daily average, maximum, and minimum temperatures, respectively, across all traps. However, comparisons between the four lure types indicated the empty mixing jars for blank lures had statistically different mean daily average, maximum, and minimum temperatures than jars containing lures (all ANOVA models, F_3,56_ ≥ 5.47, *P* ≤ 0.002). Mean daily average temperature in jars of empty, blank lures was higher at 14.9°C, compared to 14.3, 14.3 or 14.4°C in jars with lures (all *t_56_* ≥ 2.8, *P* ≤ 0.006), but no differences among the jars with lures (all *t_56_* ≤ 0.8, *P* ≥ 0.421). Mean daily maximum temperatures in empty blank jars were 24.8°C, compared to 23.2, 23.4 and 23.6°C in jars with lures (all *t_56_* ≥ 3.4, *P* < 0.002), with no differences among the jars with lures (all *t_56_* ≤ 1.1, *P* ≥ 0.268). Daily mean minimum temperatures in empty blank jars were lower at 5.0°C compared with 5.2, 5.3 and 5.3°C in jars with lures (all t_56_ ≥ 3.2, *P* < 0.002), and no differences among jars with lures (all t_56_ ≤ 1.9, *P* ≥ 0.063).
